# Development of the RMT20, a composite screener to identify common mental disorders

**DOI:** 10.1192/bjo.2020.37

**Published:** 2020-05-18

**Authors:** Philip J. Batterham, Matthew Sunderland, Natacha Carragher, Alison L. Calear

**Affiliations:** Centre for Mental Health Research, Research School of Population Health, The Australian National University, Australia; Matilda Centre for Research in Mental Health and Substance Use, Sydney Medical School, Faculty of Medicine and Health, The University of Sydney, Australia; World Health Organization, Switzerland; and Office of Medical Education, UNSW Sydney, Australia

**Keywords:** Screening, assessment, depression, anxiety

## Abstract

**Background:**

There are few very brief measures that accurately identify multiple common mental disorders.

**Aims:**

The aim of this study was to develop and assess the psychometric properties of a new composite measure to screen for five common mental disorders.

**Method:**

Two cross-sectional psychometric surveys were used to develop (*n* = 3175) and validate (*n* = 3620) the new measure, the Rapid Measurement Toolkit-20 (RMT20) against diagnostic criteria. The RMT20 was tested against a DSM-5 clinical checklist for major depression, generalised anxiety disorder, panic disorder, social anxiety disorder and post-traumatic stress disorder, with comparison with two measures of general psychological distress: the Kessler-10 and Distress Questionnaire-5.

**Results:**

The area under the curve for the RMT20 was significantly greater than for the distress measures, ranging from 0.86 to 0.92 across the five disorders. Sensitivity and specificity at prescribed cut-points were excellent, with sensitivity ranging from 0.85 to 0.93 and specificity ranging from 0.73 to 0.83 across the five disorders.

**Conclusions:**

The RMT20 outperformed two established scales assessing general psychological distress, is free to use and has low respondent burden. The measure is well-suited to clinical screening, internet-based screening and large-scale epidemiological surveys.

## Existing screening tools for common mental disorders

Mental disorders are common in the community but often go unrecognised, contributing to low rates of treatment.^1,2^ Using screening measures to identify individuals who might be experiencing a mental health problem may lead to better uptake of evidence-based treatments.^3^ Accurate screening measures may also be used to identify individuals who require more comprehensive assessment,^4,5^ used to guide the tailoring of treatment in the context of internet-based and face-to-face interventions (for example Batterham et al^6^ and Kiropoulos et al^7^), or used to assist individuals in the community to recognise whether they are likely to be experiencing a specific mental disorder.^8^ However, there are few brief measures that can be used to accurately screen for a range of common mental health problems.^8,9^

There are two broad approaches to screening for mental health problems: assessing general psychological distress and assessing the presence of specific common mental disorders. Screeners that assess general psychological distress capture internalising symptoms (i.e. symptoms of mood or anxiety disorders) at a transdiagnostic level.^10,11^ Psychological distress measures, such as the Distress Questionnaire-5 (DQ5^12^) and Kessler-6/Kessler-10 (K6/K10^13^) are accurate for identifying a range of mental disorders. However, this approach to screening does not provide direction as to which disorder is most likely to be present, may miss specific manifestations of distress, and may not cover symptoms of disorders other than major depressive disorder (MDD) or generalised anxiety disorder (GAD).^10,12^

There have been attempts to generate brief composite screeners to identify a broader spectrum of mental disorders in online, community or clinical settings.^8,9^ However, finding an appropriate balance between the brevity of the screener and its accuracy has proven challenging. Screening measures typically aim to maximise sensitivity (low rates of false negatives), so as not to miss any individuals who may meet clinical criteria for a disorder, along with adequate levels of specificity.^14^ Multidisorder composite screeners that typically consist of between two and five items per disorder have been found to perform with varying degrees of success in identifying common mental disorders in the community.^8^

## Use of item banks to assess mental disorders

Our team has recently developed item banks to assess a range of specific mental disorders, labelled as Rapid Measurement Toolkit (RMT) item banks.^15^ The RMT item banks complement the existing Patient Reported Outcomes Measurement Information System (PROMIS) emotional health measures that assess depression and anxiety.^16^ RMT and PROMIS item banks were developed through extensive multistage processes that included evaluation of items with experts and people with lived experience of mental illness, and validation in population-based samples using item-response theory methods.^15–17^ Items within these banks have been demonstrated to be free from invariance on the basis of age, gender and education. Items are also free of local dependence, that is, items are not correlated after accounting for variance in the latent construct, which makes them appropriate candidates for use in unidimensional measurement tools.^15^

By combining items from the RMT and PROMIS item banks, it may be possible to identify a parsimonious set of items that enable accurate and efficient screening for common mental disorders. However, no existing studies have assessed whether items from these dimensional measures also provide accurate screening to identify individuals who meet clinical criteria for a disorder.

## Aims

The aim of the present study was to develop a composite screener for common mental disorders and, in two population-based samples, assess its psychometric properties in identifying clinical ‘caseness’ for five common mental disorders: MDD, GAD, social anxiety disorder (SAD), panic disorder and post-traumatic stress disorder (PTSD). Two measures of general psychological distress, the DQ5 and K10, were used as comparators for identifying individuals who met criteria for each disorder, on the basis of area under the receiver operating characteristic curve (AUC). The new multidisorder screener, called the Rapid Measurement Toolkit-20 (RMT20), was designed to have high sensitivity with acceptable specificity in assessing the presence of five mental disorders. The screener was also designed using items that have been shown to be precise in assessing severity of symptoms for each of the five included disorders.^15,16^ Screeners that can also provide an indication of severity to the clinician may have higher utility than those that only assess the presence or absence of a disorder.^18,19^

## Method

### Participants and procedures

Two independent samples were recruited using virtually identical methods, separated by 13 months. These samples are referred to as the ‘development’ sample (*n* = 3175) and the ‘validation’ sample (*n* = 3620) – the screener was developed using data from the development sample^15^ then validated using data from the validation sample.^20^ All participants were recruited using advertisements on the online social media platform Facebook, with the development sample recruited between August and December 2014 and the validation sample recruited between January and February 2016. Advertisements linked directly to the survey and targeted Australian adults aged 18 years or older.

The content of the advertisement was designed to attract oversampling of people with symptoms of a mental disorder, emphasising that the research was on the topic of mental health. The survey was implemented online using Qualtrics survey software. From 39 945 individuals who clicked on the advertisement for the initial (development) survey, 10 082 (25%) consented and commenced the survey and 5011 (50%) completed the full survey, with 3175 allocated to complete all of the scales included in the present study (a short form of the survey was administered to the remaining 1836 participants). For the second (validation) survey, 5379 individuals clicked on the advertisement, 5220 (97%) consented and commenced the survey and 3620 (69%) of these completed the full survey. There were no missing data as responses were required for all questions except age and gender, with participants encouraged to discontinue if they were uncomfortable with the survey.

Written informed consent was obtained from all participants. Participants were given details of local and national mental health resources, along with the contact details of the research team, to facilitate access to mental health support if required.

The development survey included pools of items to assess SAD, panic disorder, PTSD, obsessive–compulsive disorder (OCD), adult attention-deficit hyperactivity disorder (ADHD) and substance use disorder, described previously.^15,17^ In addition, the PROMIS-depression, PROMIS-anxiety and PROMIS-alcohol use item banks^16,21^ were administered, but only in the development sample as these measures are established. A number of other existing scales related to mental health and suicide prevention were also included, but are not the focus of the present study.

Each survey took approximately 40–60 min to complete. Participants in the development survey were offered the opportunity to enter into a draw for one of four iPad Minis; no incentive was provided to participants in the validation survey. The authors assert that all procedures contributing to this work comply with the ethical standards of the relevant national and institutional committees on human experimentation and with the Helsinki Declaration of 1975, as revised in 2008. All procedures involving human patients were approved by the ANU Human Research Ethics Committee (protocols: #2013/509 and #2015/717).

### Measures

#### Item banks

The item banks used to assess MDD and GAD were the PROMIS-depression and PROMIS-anxiety item banks, whereas item banks for SAD, panic disorder and PTSD were the respective RMT item banks. All items used a first-person perspective. PROMIS item banks are rated based on the past 7 days, whereas RMT item banks are based on the past 30 days. Response to all items are on a 5-point frequency Likert scale: never (1), rarely (2), sometimes (3), often (4), always (5). The complete item banks have previously been published.^16,22^ The item banks were designed through systematic item selection and refinement processes, resulting in unidimensional, accurate measures to assess specific mental disorders.^15,16,23^ The authors of the current study developed the RMT item banks but were not involved in the development of the PROMIS item banks.

#### Clinical diagnoses

Clinical diagnoses were made using the DSM-5 symptom checklist, developed by the authors as a self-report assessment for clinical diagnosis based on DSM-5 criteria.^22,23^ The checklist queried respondents about the presence or absence of symptoms based on DSM-5 definitions for each disorder of interest. Eight items assessed SAD; 21 for panic disorder; 14 for GAD; 15 for MDD (including items to exclude hypomania); 22 for PTSD; 14 for OCD; and 21 for ADHD. Each item reflected a single DSM-5 criterion for the disorder of interest, although some criteria were probed across multiple questions and additional items were used to exclude alternative diagnoses.

Example items included: ‘In the past six months, did social situations nearly always make you feel frightened or anxious?’, ‘During the past six months, has your behaviour or difficulty in paying attention caused problems at home, work, school, or socially?’ and ‘In the past month, has there been a time when you unexpectedly felt intense fear or discomfort?’ The checklist was designed along similar principles to the electronic version of the Mini International Neuropsychiatric Interview (MINI^24^) in terms of structure (binary and categorical self-report items with conditional skip logic) and response burden. However, the checklist used in the current study was developed independently from the MINI, non-proprietary and based on DSM-5 rather than DSM-IV criteria. The full checklist has been published previously^22^ and is available from the authors.

#### Comparator scales

Comparator scales to test the relative precision of the new screener were the DQ5^12^ and K10.^13^ Both scales are accurate unidimensional measures of general psychological distress and are accurate in identifying individuals who are likely to meet clinical criteria for a range of common internalising disorders.^10,13^ The DQ5 (α = 0.91) and K10 (α = 0.94) both had excellent internal consistency in the validation sample.

#### Demographic factors

Demographic factors were collected to describe the participants and were based on self-reported measures of age group, gender (male, female, other), educational attainment, employment status, location (metropolitan, regional, rural) and language spoken at home.

### Analysis

From the five item banks (PROMIS-depression, PROMIS-anxiety, RMT social anxiety, RMT panic, RMT PTSD), four items for each disorder were chosen on the basis of their accuracy in assessing clinical criteria for the disorder of interest. These 20 items formed the RMT20 screener. We initially tested screeners with 3–6 items for each disorder but found that adding items beyond four typically did not substantially improve the sensitivity and specificity of the screener within the development sample. The items were chosen to provide coverage across the spectrum of severity that is measured by the full item banks.^25^ Specifically, items were selected based on item response theory (IRT) discrimination and difficulty parameters as well as item information curves when measuring a single unidimensional construct representing either panic disorder, SAD or PTSD.^25^ This approach ensured that the screeners were accurate across the continuum of the latent construct.

The PROMIS screeners were only administered within the development sample, as they are established item banks, whereas screeners for the RMT measures were then tested in the validation sample. The AUC was the indicator of the precision of each subscreener. AUC for the new subscreeners were compared to AUC for the DQ5 and K10 for each of the five disorders.

Cut-points were defined based on Youden indices, although with a view to maximising sensitivity when there were comparable choices. Sensitivity and specificity (with 95% CI) were estimated at each cut-point and compared with the sensitivity and specificity of the DQ5 and K10 based on prescribed cut-points.^12^

## Results

The characteristics of the two samples are provided in Table 1. There were significant differences in all variables except for language, GAD caseness, panic disorder caseness, PTSD caseness and K10 score. Participants in the validation sample appeared to be slightly younger, better educated, had higher employment rates, and a greater proportion resided in urban areas. In addition, the validation sample had a lower prevalence of depression and SAD and less severe distress than the development sample. Nevertheless, differences were typically small (for example Cohen's *d* = 0.12 for DQ5), which suggests that the statistical significance of these comparisons may be more related to sample size than clinically meaningful differences.

**Table 1:** Characteristics of the development and validation samples

	Development sample (*n* = 3175)		Validation sample (*n* = 3620)				
	*n*	(%)	*n*	(%)	*χ^2^*	*t*	*P*
Age group, *n* (%)					129.49		**<0**.**001**
18–25 years	371	(11.7)	718	(19.8)			
26–35 years	277	(8.7)	443	(12.2)			
36–45 years	506	(15.9)	539	(14.9)			
46–55 years	830	(26.1)	738	(20.4)			
56–65 years	842	(26.5)	815	(22.5)			
66 years or older	349	(11.0)	365	(10.1)			
Prefer not to answer	0	(0.0)	2	(0.1)			
Gender, *n* (%)					37.60		**<0**.**001**
Male	648	(20.4)	689	(19.0)			
Female	2527	(79.6)	2890	(79.8)			
Other	0	(0.0)	30	(0.8)			
Prefer not to answer	0	(0.0)	11	(0.3)			
Education, *n* (%)					145.47		**<0**.**001**
High school or less	382	(12.0)	254	(7.0)			
High school graduate	440	(13.9)	528	(14.6)			
Certificate/diploma/associate degree	963	(30.3)	803	(22.2)			
Bachelor degree	630	(19.8)	904	(25.0)			
Postgraduate degree	747	(23.5)	1123	(31.0)			
Prefer not to answer	13	(0.4)	8	(0.2)			
Employment status, *n* (%)					27.88		**<0**.**001**
Full time	856	(27.0)	1054	(29.1)			
Part time/casual	819	(25.8)	1067	(29.5)			
Unemployed	383	(12.1)	430	(11.9)			
Not working (study/leave/retired)	1070	(33.7)	1024	(28.3)			
Prefer not to answer	47	(1.5)	45	(1.2)			
Location, *n* (%)					41.31		**<0**.**001**
Metropolitan area (capital city)	1421	(44.8)	1891	(52.2)			
Regional area (other city/town)	1279	(40.3)	1308	(36.1)			
Rural or remote area	475	(15.0)	421	(11.6)			
Language spoken at home, *n* (%)					2.62		0.270
English only	2962	(93.3)	3344	(92.4)			
English and another language	199	(6.3)	262	(7.2)			
Another language only	14	(0.4)	14	(0.4)			
Met clinical criteria for MDD, *n* (%)	362	(11.4)	309	(8.5)	15.61		**<0**.**001**
Met clinical criteria for GAD, *n* (%)	645	(20.3)	805	(22.2)	3.73		0.054
Met clinical criteria for SAD, *n* (%)	551	(17.4)	529	(14.6)	9.51		**0**.**002**
Met clinical criteria for panic disorder, *n* (%)	186	(5.9)	190	(5.2)	1.20		0.273
Met clinical criteria for PTSD, *n* (%)	391	(12.3)	410	(11.3)	1.59		0.207
Met criteria for any of these disorders, *n* (%)	1135	(35.7)	1229	(34.0)	2.33		0.127
Distress Questionnaire-5, mean (s.d.)	11.48	(5.10)	10.86	(4.81)		5.16	**<0**.**001**
Kessler-10, mean (s.d.)	11.90	(8.90)	11.86	(8.79)		0.21	0.835

MDD, major depressive disorder; GAD, generalised anxiety disorder; SAD: social anxiety disorder; PTSD: post-traumatic stress disorder.

Bold values indicate *P* < 0.05.

The eight PROMIS items and 12 RMT items selected for the final composite screener (RMT20) are provided in Table 2, including mean (s.d.) for individuals with and without the specific disorder of interest. It should be noted that these means are likely higher than would be seen in the general population, so should not be considered normative data. All items significantly differentiated those with and without the disorder of interest.

**Table 2:** Mean (s.d.) and 95% CI for items included in the composite screener, based on presence or absence of the disorder of interest

	Disorder of interest, absent			Disorder of interest, present		
*MDD, n*	2813			363		
MDD subscreener, mean (s.d.) 95% CI						
I felt depressed	2.34	(1.17)	2.30–2.39	4.22	(0.75)	4.14–4.29
I felt hopeless	2.02	(1.14)	1.97–2.06	3.94	(0.92)	3.84–4.03
I felt that nothing could cheer me up	1.85	(1.10)	1.81–1.89	3.83	(0.91)	3.73–3.92
I felt that I had nothing to look forward to	2.00	(1.19)	1.96–2.05	3.98	(0.94)	3.89–4.08
*GAD, n*	2530			645		
GAD subscreener, mean (s.d.) 95% CI						
I felt anxious	2.17	(1.02)	2.13–2.21	3.63	(0.91)	3.56–3.70
I felt worried	2.23	(1.00)	2.19–2.27	3.65	(0.85)	3.59–3.72
My worries overwhelmed me	1.86	(1.01)	1.82–1.90	3.42	(0.97)	3.35–3.50
Many situations made me worry	1.91	(1.03)	1.87–1.95	3.46	(0.97)	3.39–3.54
*SAD, n*	3091			529		
SAD subscreener, mean (s.d.) 95% CI						
I felt nervous during social situations	2.44	(1.08)	2.40–2.48	4.09	(0.75)	4.03–4.15
My fear of social situations was distressing	1.80	(1.00)	1.76–1.84	3.69	(0.95)	3.61–3.77
I was unable to relax during social situations	2.15	(1.07)	2.12–2.19	3.85	(0.89)	3.78–3.93
I felt tense about mixing in a group	2.29	(1.10)	2.25–2.33	4.01	(0.87)	3.93–4.08
*Panic disorder, n*	3430			190		
Panic disorder subscreener, mean (s.d.) 95% CI						
I felt afraid of certain activities because I feared having a panic attack	1.64	(0.97)	1.61–1.67	3.05	(1.12)	2.89–3.21
I had a sudden unexpected period of intense fear, anxiety or discomfort	2.06	(1.06)	2.02–2.09	3.44	(0.86)	3.32–3.56
I felt terrified, my heart raced and I thought I might be dying	1.42	(0.78)	1.40–1.45	2.68	(1.04)	2.53–2.83
I worried about having an unexpected anxiety spell or panic attack	1.65	(0.99)	1.62–1.68	3.10	(1.08)	2.95–3.25
*PTSD, n*	3210			410		
PTSD subscreener, mean (s.d.) 95% CI						
I could not feel close to others because of a trauma	1.51	(0.91)	1.48–1.54	3.11	(1.23)	2.99–3.23
I felt cut off and isolated from other people because of a trauma	1.53	(0.94)	1.50–1.56	3.23	(1.26)	3.11–3.35
I did not enjoy the company of others because of a trauma	1.45	(0.86)	1.42–1.48	2.99	(1.25)	2.87–3.11
I felt on edge and distressed when thinking about a trauma	1.63	(1.00)	1.60–1.66	3.37	(1.24)	3.25–3.49

MDD, major depressive disorder; GAD, generalised anxiety disorder; SAD: social anxiety disorder; PTSD: post-traumatic stress disorder.

Table 3 details the precision of the subscreeners in identifying clinical caseness for the five mental disorders based on AUC, with comparison with the two measures of psychological distress: DQ5 and K10. The table indicates that the disorder-specific subscreeners from the RMT20 were significantly more precise in screening for all disorders of interest, except in the case of GAD where the difference between the RMT20 and DQ5 was not significant. For SAD and panic disorder in particular, the RMT20 had considerably greater precision than both the DQ5 and K10, with an increase of up to 9% in AUC.

**Table 3:** Comparison of area under the curve for new 4-item subscreeners in comparison with existing measures of general psychological distress^a^

Disorder	RMT20 subscreener AUC (95% CI)	K10 AUC (95% CI)	DQ5 AUC (95% CI)	RMT20 *v*ersus K10		RMT20 *v*ersus DQ5	
				*χ^2^*	*P*	*χ^2^*	*P*
Major depression (D)	0.917 (0.905–0.930)	0.900 (0.887–0.913)	0.898 (0.885–0.912)	6.88	**0**.**009**	8.65	**0**.**003**
Generalised anxiety (D)	0.889 (0.876–0.901)	0.849 (0.835–0.864)	0.880 (0.867–0.894)	32.57	**<0**.**001**	2.72	0.099
Social anxiety (V)	0.916 (0.905–0.928)	0.844 (0.827–0.861)	0.868 (0.853–0.883)	94.11	**<0**.**001**	57.00	**<0**.**001**
Panic disorder (V)	0.878 (0.859–0.896)	0.807 (0.780–0.835)	0.810 (0.783–0.837)	35.63	**<0**.**001**	38.38	**<0**.**001**
PTSD (V)	0.863 (0.844–0.883)	0.820 (0.800–0.839)	0.815 (0.796–0.835)	14.18	**<0**.**001**	17.43	**<0**.**001**

(D), data from the development sample (*n* = 3175); (V), data from the validation sample (*n* = 3620); PTSD: post-traumatic stress disorder; AUC, area under the receiver operating characteristic curve; K10, Kessler-10; DQ5, Distress Questionnaire-5.

a. *χ^2^* tests have one degree of freedom; bold values indicate *P* < 0.05.

Table 4 shows the performance of the subscreeners at the identified cut-points. All screeners had high sensitivity, approximately 85% or greater, and specificity above 70%, indicating their accuracy across independent samples. The subscreeners also had high internal consistency, at or above 0.9. Performance of the RMT20 was stronger overall than the distress screeners, with similar or high sensitivity and specificity at prescribed cut-points.

**Table 4:** Performance of the subscreeners at selected cut-points

	RMT20						K10 ≥ 25		DQ5 ≥ 14	
Disorder	Items	Range	Cronbach α	Cut-point	Sensitivity (95% CI)	Specificity (95% CI)	Sensitivity (95% CI)	Specificity (95% CI)	Sensitivity (95% CI)	Specificity (95% CI)
Major depression (D)	4	4–20	0.944	≥13	89.5 (85.9–92.5)	82.6 (81.2–84.0)	92.8 (89.7–95.3)	71.5 (69.8–73.1)	86.7 (82.8–90.0)	78.2 (76.6–79.7)
Generalised anxiety (D)	4	4–20	0.925	≥11	88.6 (86.0–91.0)	73.4 (71.6–75.1)	79.5 (76.2–82.6)	75.3 (73.5–76.9)	76.9 (73.5–80.1)	82.9 (81.4–84.4)
Social anxiety (V)	4	4–20	0.930	≥12	93.2 (90.7–95.2)	76.5 (75.0–78.0)	80.7 (77.1–84.0)	73.3 (71.7–74.9)	83.4 (79.9–86.4)	73.6 (72.0–75.2)
Panic disorder (V)	4	4–20	0.891	≥9	87.4 (81.8–91.7)	74.6 (73.1–76.0)	76.8 (70.2–82.6)	67.8 (66.2–69.3)	83.1 (77.1–88.2)	68.0 (66.4–69.5)
PTSD (V)	4	4–20	0.959	≥8	84.6 (80.8–88.0)	74.4 (72.8–75.9)	78.3 (74.0–82.2)	71.0 (69.4–72.6)	77.6 (73.2–81.5)	70.8 (69.1–72.3)

(D), based on data from the development sample (*n* = 3175); (V), based on data from the validation sample (*n* = 3620); PTSD: post-traumatic stress disorder.

## Discussion

### Main findings

The psychometric properties of the RMT20 composite screening measure suggest it provides an accurate and rapid method to identify individuals who meet clinical criteria for specific common mental disorders within the general population. The RMT20 screener outperformed two established measures of general psychological distress across all disorders, suggesting the composite screener approach may be more effective and efficient when there is a need to identify the presence of specific mental disorders. The gains in precision were most evident for social anxiety, panic disorder and PTSD, which are not typically captured as well as depression and generalised anxiety by measures of general psychological distress.^10^ The RMT20 was designed for online use but may also have relevance in a range of clinical settings where identifying specific forms of psychopathology would be beneficial.

Measures of general psychological distress remain useful for identifying individuals who are likely to meet clinical criteria for one or more mental disorders. Indeed, measures such as the DQ5 have been shown to be highly accurate and efficient in screening for a range of mental disorders, using only five items. However, distress measures are unable to differentiate the specific disorder that an individual is most likely to be experiencing. The 20-item composite screener presented here provides a compromise between distress measures and lengthy batteries of mental health measures. For example, using common existing measures assessing five disorders included in the composite screener would require presentation of at least 34 items from five scales, each with different stems and response frames. The RMT20 also allows flexibility in the scope of screening, enabling the subscreeners to be administered as needed.

### Administration of the RMT20

The RMT20 performed well in relation to other brief composite screeners tested previously,^8,9^ which typically perform better for some disorders (for example MDD) than others (for example GAD, PTSD). The RMT20 also performed similarly to computer adaptive tests, which typically require 4–15 items per domain to deliver a similar level of accuracy.^26–28^ Computer adaptive tests, although similarly efficient to brief composite screeners, require considerable infrastructure to administer and do not appear to provide a considerable benefit in terms of markedly greater precision for identifying specific mental disorders in the community.^25^ In contrast, the RMT20 is easy to administer in an online or paper-and-pencil settings, with minimal loss of diagnostic accuracy.

### Potential applications

While our main focus in the development of this composite screener was on tailoring internet interventions to prevent and treat mental disorders in the community,^6^ further potential applications are extensive. Poor recognition of mental disorders in the community^29^ could be improved by provision of screening tools to the public, in conjunction with feedback to support appropriate help-seeking. Screening programmes within general practice, hospital settings and community-based mental health services often require brief and accurate indicators for a range of specific mental health problems. As a result of time constraints and consideration of patient burden, healthcare settings typically screen using psychological distress measures that may be suboptimal, or focus only on depression and/or generalised anxiety. The current findings suggest that distress screeners may not be as accurate as a composite screener in identifying particular common mental disorders such as panic disorder and PTSD. The present composite screener may provide an alternative approach to screening that is more accurate and can be administered in 1–2 min. Although the current population sample reported high levels of psychopathology, further investigation of the performance of the RMT20 in clinical settings would be warranted.

### Strengths and limitations

This study used separate development and validation data-sets to establish the psychometric properties of the new composite screener. Both data-sets consisted of large samples of adults recruited from the community, with oversampling of people with mental health problems. However, there are some limitations of the present research. First, the RMT20 did not include externalising disorders or less common internalising disorders, although there is scope to add modules for these domains in future. The sample was not representative of the population nor of a treatment-seeking clinical sample, so further data may be needed to provide population norms. The clinical outcome measure was a self-report checklist, which is similar to the approach used in other population-based studies. Such checklists provide an indication for probable disorder only, so further evaluation of the RMT20 against a clinician diagnosis would be valuable. Furthermore, the development of the RMT20 was on the basis of accuracy in assessing DSM-5 disorders, so some degree of circularity in the definitions used in RMT20 may exist, excluding broader manifestations of these disorders. Although psychological distress scales are widely used for screening, a stronger future comparison for the RMT20 may be against a battery of more traditional disorder-specific screening measures (see for example Zuromski et al,^30^ Kroenke et al^31^ and Batterham et al^32,33^). The PROMIS measures were only included in the development sample, as they are established measures, precluding us from measuring their psychometric properties across independent data-sets.

Finally, the method for selecting items was designed to maximise the accuracy of the screener across the dimensions of each disorder, rather than using methods to maximise classification, such as decision trees. The dual-function screeners may therefore be used in assessing both severity and presence of disorder. However, alternative methods for identifying subsets of items may have provided greater accuracy in capturing diagnostic criteria.

### Implications

The RMT20 is a composite measure that is accurate in screening for five mental disorders in the community. The measure outperforms two established scales assessing general psychological distress, is free to use and is associated with low respondent burden, which makes it well-suited to busy clinical settings, internet-based screening and large-scale epidemiological surveys.

## Data Availability

All authors had full access to the study data. P.J.B. is the data custodian for the study and led the analyses. Data are available on request from P.J.B.

## References

[1] Wang PS, Berglund P, Olfson M, Pincus HA, Wells KB, Kessler RC. Failure and delay in initial treatment contact after first onset of mental disorders in the National Comorbidity Survey Replication. Arch Gen Psychiatry 2005; 62: 603–13. 15939838 10.1001/archpsyc.62.6.603

[2] Wittchen HU, Kessler RC, Beesdo K, Krause P, Hofler M, Hoyer J. Generalized anxiety and depression in primary care: prevalence, recognition, and management. J Clin Psychiatry 2002; 63 (Suppl 8): 24–34. 12044105

[3] Hirschfeld RM. The comorbidity of major depression and anxiety disorders: recognition and management in primary care. Prim Care Companion J Clin Psychiatry 2001; 3: 244–54. 15014592 10.4088/pcc.v03n0609 PMC181193

[4] Sen B, Wilkinson G, Mari JJ. Psychiatric morbidity in primary health care. A two-stage screening procedure in developing countries: choice of instruments and cost-effectiveness. Br J Psychiatry 1987; 151: 33–8. 3118997 10.1192/bjp.151.1.33

[5] Scott MA, Wilcox HC, Schonfeld IS, Davies M, Hicks RC, Turner JB, School-based screening to identify at-risk students not already known to school professionals: the Columbia suicide screen. Am J Public Health 2009; 99: 334–9. 19059865 10.2105/AJPH.2007.127928 PMC2622792

[6] Batterham PJ, Calear AL, Farrer L, McCallum SM, Cheng VWS. FitMindKit: randomised controlled trial of an automatically tailored online program for mood, anxiety, substance use and suicidality. Internet Interv 2018; 12: 91–9. 30135773 10.1016/j.invent.2017.08.002 PMC6096326

[7] Kiropoulos LA, Kilpatrick T, Holmes A, Threader J. A pilot randomized controlled trial of a tailored cognitive behavioural therapy based intervention for depressive symptoms in those newly diagnosed with multiple sclerosis. BMC Psychiatry 2016; 16: 435. 27927175 10.1186/s12888-016-1152-7 PMC5142334

[8] Donker T, van Straten A, Marks I, Cuijpers P. A brief web-based screening questionnaire for common mental disorders: development and validation. J Med Internet Res 2009; 11: e19. 19632977 10.2196/jmir.1134 PMC2763401

[9] Farvolden P, McBride C, Bagby RM, Ravitz P. A web-based screening instrument for depression and anxiety disorders in primary care. J Med Internet Res 2003; 5: e23. 14517114 10.2196/jmir.5.3.e23 PMC1550568

[10] Batterham PJ, Sunderland M, Slade T, Calear AL, Carragher N. Assessing distress in the community: psychometric properties and crosswalk comparison of eight measures of psychological distress. Psychol Med 2018; 48: 1316–24. 28967345 10.1017/S0033291717002835

[11] Sunderland M, Batterham P, Carragher N, Calear A, Slade T. Developing and validating a computerized adaptive test to measure broad and specific factors of internalizing in a community sample. Assessment 2019; 26: 1030–45. 28467115 10.1177/1073191117707817

[12] Batterham PJ, Sunderland M, Carragher N, Calear AL, Mackinnon AJ, Slade T. The Distress Questionnaire-5: population screener for psychological distress was more accurate than the K6/K10. J Clin Epidemiol 2016; 71: 35–42. 26464194 10.1016/j.jclinepi.2015.10.005

[13] Kessler RC, Andrews G, Colpe LJ, Hiripi E, Mroczek DK, Normand SL, Short screening scales to monitor population prevalences and trends in non-specific psychological distress. Psychol Med 2002; 32: 959–76. 12214795 10.1017/s0033291702006074

[14] Zarin DA, Earls F. Diagnostic decision making in psychiatry. Am J Psychiatry 1993; 150: 197–206. 8422070 10.1176/ajp.150.2.197

[15] Batterham PJ, Sunderland M, Carragher N, Calear AL. Development and community-based validation of eight item banks to assess mental health. Psychiatry Res 2016; 243: 453–62. 27500552 10.1016/j.psychres.2016.07.011

[16] Pilkonis PA, Choi SW, Reise SP, Stover AM, Riley WT, Cella D. Item banks for measuring emotional distress from the Patient-Reported Outcomes Measurement Information System (PROMIS(R)): depression, anxiety, and anger. Assessment 2011; 18: 263–83. 21697139 10.1177/1073191111411667 PMC3153635

[17] Batterham PJ, Brewer JL, Tjhin A, Sunderland M, Carragher N, Calear AL. Systematic item selection process applied to developing item pools for assessing multiple mental health problems. J Clin Epidemiol 2015; 68: 913–9. 25953659 10.1016/j.jclinepi.2015.03.022

[18] Kessler RC. The categorical versus dimensional assessment controversy in the sociology of mental illness. J Health Soc Behav 2002; 43: 171–88. 12096698

[19] Andrews G, Anderson TM, Slade T, Sunderland M. Classification of anxiety and depressive disorders: problems and solutions. Depress Anxiety 2008; 25: 274–81. 18415950 10.1002/da.20489

[20] Batterham PJ, Calear AL, Carragher N, Sunderland M. Prevalence and predictors of distress associated with completion of an online survey assessing mental health and suicidality in the community. Psychiatry Res 2018; 262: 348–50. 28843625 10.1016/j.psychres.2017.08.048

[21] Pilkonis PA, Yu L, Dodds NE, Johnston KL, Lawrence SM, Daley DC. Validation of the alcohol use item banks from the Patient-Reported Outcomes Measurement Information System (PROMIS). Drug Alcohol Depend 2016; 161: 316–22. 26936412 10.1016/j.drugalcdep.2016.02.014 PMC6032515

[22] Batterham PJ, Calear AL, Christensen H, Carragher N, Sunderland M. Independent effects of mental disorders on suicidal behavior in the community. Suicide Life Threat Behav 2018; 48: 512–21. 28782126 10.1111/sltb.12379

[23] Batterham PJ, Sunderland M, Carragher N, Calear AL. Psychometric properties of 7- and 30-day versions of the PROMIS emotional distress item banks in an Australian adult sample. Assessment 2019; 26: 249–59. 28052687 10.1177/1073191116685809

[24] Zbozinek TD, Rose RD, Wolitzky-Taylor KB, Sherbourne C, Sullivan G, Stein MB, Diagnostic overlap of generalized anxiety disorder and major depressive disorder in a primary care sample. Depress Anxiety 2012; 29: 1065–71. 23184657 10.1002/da.22026 PMC3629816

[25] Sunderland M, Batterham PJ, Calear AL, Carragher N. The development and validation of static and adaptive screeners to measure the severity of panic disorder, social anxiety disorder, and obsessive compulsive disorder. Int J Methods Psychiatr Res 2017; 26: e1561. 10.1002/mpr.1561 PMC6877206 28370638

[26] Graham AK, Minc A, Staab E, Beiser DG, Gibbons RD, Laiteerapong N. Validation of the computerized adaptive test for mental health in primary care. Ann Fam Med 2019; 17: 23–30. 30670391 10.1370/afm.2316 PMC6342585

[27] Gibbons RD, Weiss DJ, Pilkonis PA, Frank E, Moore T, Kim JB, Development of a computerized adaptive test for depression. Arch Gen Psychiatry 2012; 69: 1104–12. 23117634 10.1001/archgenpsychiatry.2012.14 PMC3551289

[28] Gibbons RD, Weiss DJ, Pilkonis PA, Frank E, Moore T, Kim JB, Development of the CAT-ANX: a computerized adaptive test for anxiety. Am J Psychiatry 2014; 171: 187–94. 23929270 10.1176/appi.ajp.2013.13020178 PMC4052830

[29] Reavley NJ, Jorm AF. Recognition of mental disorders and beliefs about treatment and outcome: findings from an Australian national survey of mental health literacy and stigma. Aust N Z J Psychiatry 2011; 45: 947–56. 21995330 10.3109/00048674.2011.621060

[30] Zuromski KL, Ustun B, Hwang I, Keane TM, Marx BP, Stein MB, Developing an optimal short-form of the PTSD Checklist for DSM-5 (PCL-5). Depress Anxiety 2019; 36: 790–800. 31356709 10.1002/da.22942 PMC6736721

[31] Kroenke K, Spitzer RL, Williams JB, Lowe B. An ultra-brief screening scale for anxiety and depression: the PHQ-4. Psychosomatics 2009; 50: 613–21. 19996233 10.1176/appi.psy.50.6.613

[32] Batterham PJ, Mackinnon AJ, Christensen H. Community-based validation of the Social Phobia Screener (SOPHS). Assessment 2017; 24: 958–69. 26939801 10.1177/1073191116636448

[33] Batterham PJ, Mackinnon AJ, Christensen H. The panic disorder screener (PADIS): development of an accurate and brief population screening tool. Psychiatry Res 2015; 228: 72–6. 25956758 10.1016/j.psychres.2015.04.016

